# Reducing scratching behavior in atopic dermatitis patients using the EMDR treatment protocol for urge: A pilot study

**DOI:** 10.3389/fmed.2023.1101935

**Published:** 2023-04-04

**Authors:** Mathijs R. de Veer, Rick Waalboer-Spuij, Dirk Jan Hijnen, Do Doeksen, Jan J. Busschbach, Leonieke W. Kranenburg

**Affiliations:** ^1^Department of Psychiatry, Section Medical Psychology, Erasmus University Medical Centre, Rotterdam, Netherlands; ^2^Department of Dermatology, Erasmus University Medical Centre, Rotterdam, Netherlands; ^3^Private Practice in Psychotherapy, Delft, Netherlands

**Keywords:** atopic dermatitis, itch, scratching, Eye Movement Desensitization and Reprocessing (EMDR), urge, DEP

## Abstract

**Background:**

Itch, and thereby the scratching behavior, is a common complaint in atopic dermatitis. Scratching damages the skin, which in turn worsens the itch. This itch-scratch cycle perpetuates the skin condition and has a major impact on the patient's quality of life. In addition to pharmacological treatment, psychological interventions show promising results in reducing scratching behavior.

**Objectives:**

To investigate the effect of treatment according the EMDR treatment protocol for urge on scratching behavior of atopic dermatitis patients in a controlled study.

**Methods:**

This study applies a multiple baseline across subjects design. Six patients were randomly allocated to different baseline lengths and all of them started registration of scratching behavior at the same day, using a mobile phone application. Nocturnal scratching was registered by a smart watch application. The total study duration was 46 days and was equal for all patients. Treatment consisted of two sessions using the EMDR treatment protocol for urge. Furthermore, standardized measures were used to assess disease activity, quality of life, and self-control. The nonoverlap of all pairs effect size was calculated for the daily measure data.

**Results:**

One patient dropped out. Visual inspection suggests that the scratching behavior decreased over time in all patients. Furthermore, a moderate effect size of the treatment is found. During the baseline phase, scratching behavior fluctuated considerably and showed a slight negative trend. Outcomes of disease activity decreased over time and patients' self-control and quality of life improved after treatment. Nocturnal scratching behavior did not change after the intervention.

**Conclusion:**

The results of the visual analysis of day time scratching behavior, disease activity, quality of life, and self-control seem promising. These findings pave the way for future research into the effect of the new intervention on other skin conditions suffering from scratching behavior, such as prurigo nodularis.

## Introduction

Atopic dermatitis (AD) occurs in ~1 to 10 % of all adults (1, 2) and is characterized by chronic inflammation of the skin. Skin inflammation results in itch, which results in scratching, and a negative feedback loop causing worsening of the skin condition. Itch has been found to drive the burden of AD, as it causes sleeping problems and is related to reports of pain, anxiety and depression (3). Mental health scores for AD patients were described lower than those of patients with other chronic health conditions such as diabetes and heart diseases (4). The disease and the more or less continuous itch severely impact patients' daily and working lives, and their health-related quality of life (3, 5), and asks for a multidisciplinary approach (6).

Besides pharmacological treatment, psychological interventions that target scratching behavior show significant ameliorating effects on itching intensity and scratching (7, 8). Psychological treatments for scratching behavior are based on ‘self-control procedures' and ‘habit reversal'. More recent, novel types of treatment to reduce scratching behavior in AD patients appear to be effective, such as internet-delivered and exposure-based cognitive behavioral therapy (9, 10) and a self-care intervention without therapist support (11).

The psychological intervention to be investigated in this study is the EMDR treatment protocol for urge (*Drang EMDR Protocol*, DEP; Doeksen, 2018) (12), which draws on elements of Eye Movement Desensitization and Reprocessing (EMDR) therapy, cognitive behavioral therapy, and hypnotherapy. In the current treatment, not the full EMDR procedure is applied, but only the EMD-part—that is the desensitization part. Desensitization aims at the “fading out” or “losing urge” for the behavior that is longed for, in this case the scratching (13). Patients are allowed to perform the scratching in imagination, while at the same time the working memory is being taxed. The use of EMDR to alter addictive behavior has been previously studied by Popky (14). In 2005, Popky introduced an Urge Reduction Protocol for Addictions and Dysfunctional Behaviors (DeTUR). DeTUR consists of multiple steps, including positive goal setting, and the identification and desensitization of triggers of the unwanted behavior. Moreover, clients learn to use the technique at home. DeTUR was shown to be effective in reducing unwanted behavior in multiple case studies in clients with substance use disorders, eating disorders and trichotillomania. Furthermore, the DEP treatment protocol draws on elements of cognitive behavior therapy, as self-registration of behavior and homework assignments are core elements of treatment. Finally, elements of hypnotherapy are incorporated in this treatment, with respect to the interpretation to perceive the treated skin spots—that does (no longer) evokes the urge to scratch—as “calm and white.” This protocol turned out to be successful in a number of individual treatments (12), but the intervention has not been subject of scientific research yet. Therefore, we aim to investigate the effects of this intervention in a controlled study.

## Materials and methods

### Participants

Patients with a confirmed diagnosis of AD and systemic immunosuppressive treatment were included. Only patients with stable disease activity, suffering from persistent and frequent scratching behavior, no successful response to care as usual, and sufficiently motivated to take part in a new intervention aimed at behavior change, were eligible for study participation. Patients were invited to participate in the study by their treating dermatologist. All patients signed informed consent. The study was approved by the medical scientific research Ethical Committee of the Erasmus University Medical Center (reference number MEC-2020-0127).

### Study design and intervention

**Figure 1:**
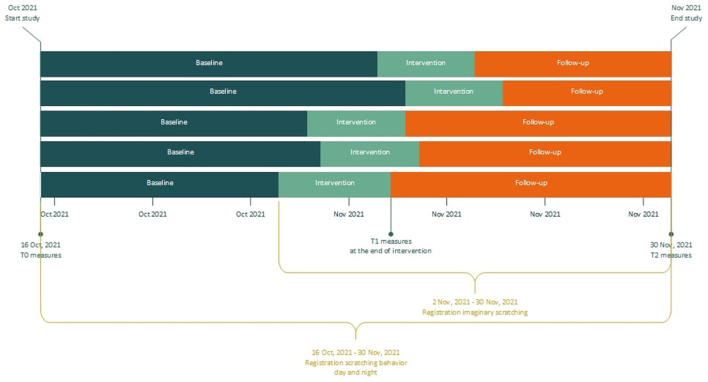
Flowchart of study design.

This pilot study applies a multiple baseline across subjects design, consisting of three phases: baseline, intervention and follow-up (see Figure 1, and the paragraph below). The total study duration was 46 days and was equal for all patients. All of them started registration of scratching behavior at the same day. Six participants were randomly allocated to different baseline lengths, to determine whether any observed changes in scratching behavior are due to the intervention or simply the passage of time. This randomization was not blinded, as patients and researchers knew when the treatment started. Pairs of two patients were randomly selected and were assigned to one of three possible starting weeks, with a randomly selected weekday for each patient to start treatment. Randomization was performed with a randomization application, in which the possible starting points for all six patients were entered (15). The intervention phase duration was 10 days for all patients, and consisted of two treatment sessions in the setting of the psychiatry outpatient clinic of the Erasmus University Medical Center and two additional phone calls. The intervention phase was followed by a follow-up phase.

*Baseline phase*: Patients were invited for a first meeting after signing informed consent. During this appointment they were instructed regarding the daily registration, the T0 questionnaires were administered, and the disease activity was evaluated by a dermatologist. The mobile phone application for daily registration of scratching behavior was installed on patients' mobile phone and explained. A smartwatch and mobile phone to pair the watch for night-registrations were handed out. During baseline, patients registered scratching behavior, but did not receive treatment yet.

*Intervention phase*: The EMDR treatment protocol for urge (*Drang EMDR Protocol*, DEP; Supplementary material S1) was performed by a trained EMDR Europe Accredited Practitioner supervised by a EMDR Europe Accredited Consultant, who is also the developer of the protocol (12). The intervention consisted of two DEP treatment sessions of maximum 90 min, which took place in two consecutive weeks (one session per week). Within 3 days after each session, participants were called by the therapist to discuss potential difficulties in applying the intervention at home. At the end of the intervention phase, questionnaires were administered again for T1.

*Follow-up phase*: The follow-up phase commenced after the intervention phase, and consisted of at home practice of the techniques acquired during the intervention phase. At the end of the follow-up phase, patients filled out the questionnaires for the third and last time. This last measurement took place in the hospital, as their skin was evaluated again by the dermatologist. In addition, the course of the study and the intervention were evaluated together with the research assistant, and the smartwatch and mobile phone were handed in.

During the study, patients were occasionally called by the research assistant to check if technical problems occurred, and to resolve registration issues early. The contact details of this researcher were given to the patients, so that patients had the opportunity to easily contact the researcher themselves in case of technical problems.

### Measures

In multiple baseline designs it is common to work with “target measures” and “standardized measures”. Target measures are aimed at frequent (often daily) measuring of the complaints or behavior that is to be altered (“targeted”) by the intervention under study. Standardized measures are well- validated outcome measures, that are applied at set times during the study. The aim of applying standardized measures is to get an overall idea of respondents' outcomes on well-known measures.

#### Target measures

##### Day time scratching

Frequency and duration of actual scratching behavior were measured on a daily basis. A mobile telephone application was designed for this study to register the actual scratching behavior. Each time a patient had scratched, he had to record the duration of scratching in a “hit list.” Duration was classified into seven categories (<1 min; 1–3 min; 4–5 min; 6–10 min; 11–15 min; 16–30 min; >30 min). During data analysis this was reduced to 3 categories (<1 min “short”; 1–3 min “medium”; >3 min “long”). The “day time scratching” outcome was calculated by multiplying the number of scratching episodes with duration (1 = short, 2 = medium, and 3 = long). For example, if a patient had eight short, three medium and two long scratching episodes during a day, the sum score was 20 (8 + 6 + 6).

##### Imaginary scratching

Each time a patient applied the learned intervention at home, he had to record the duration of the imaginary scratching. The number of these episodes and their duration were also registered with the mobile phone app.

##### Nocturnal scratching

Duration and intensity of scratching behavior during the night was registered by a smart watch application, developed by the Center for Human Drug Research (CHDR) (16). The outcome was the sum of the episodes of scratching, which were described as the intensity multiplied with the duration of the episode.

#### Standardized measures

*Disease activity, measure to be filled out by dermatologist at T0 and T2:* Eczema Area and Severity Index (EASI) (17). A validated scoring system that grades the physical signs of atopic dermatitis/eczema.

*Three Quality of Life measures, at T0, T1 and T2:* (1) the Patient-Oriented Eczema Measure (POEM) is a self-report questionnaire consisting of 7 items to be scored on a 4-point Likert scale (18); (2) The SKINDEX-17 is a dermatology-specific health-related quality of life (HRQOL) instrument. It consists of 17 items to be scored on a 5-point Likert scale. The instrument has two subscales: psychosocial impact and impact of symptoms (19); (3) The EQ-5D-5L measures health-related quality of life. It is a generic instrument that can be used in a wide range of health conditions and treatments. The EQ-5D-5L consists of a descriptive system and the EQ VAS. The descriptive system comprises five dimensions: mobility, self-care, usual activities, pain/discomfort and anxiety/depression. The EQ VAS records the patient's self-rated health on a vertical visual analog scale (20).

*Self-Control, at T0, T1 and T2:* The Self-Control Cognition Questionnaire, Dutch: Zelfcontrole Cognitie Vragenlijst (ZCCL). The ZCCL is an 11-item self-report questionnaire measuring perceived self-control. There are two subscales: ‘positive reward' (of the unwanted behavior) and ‘difficulty resisting'. Each item is scored on a 5-point Likert scale (21).

### Statistical analysis

Day time scratching data is analyzed by visual inspection. Moreover, the nonoverlap of all pairs (NAP) effect size is calculated, using the computer program Shiny SCDA (Single-Case Data Analysis) (15, 22). NAP, which is an index of data overlap between phases in single-case research, depends on the expected direction of the treatment effect, in this case a reduction of the scratching behavior (23). NAP is defined in terms of all pair-wise comparisons between the data points in different phases. The Shiny application can compare only two phases. We therefore combined the intervention and follow-up phase and compared those combined phases with the baseline phase.

Standardized measures were analyzed using IBM SPSS Statistics for Windows, Version 28.0 was used (Armonk, NY: IBM Corp) and are presented as descriptives, as no statistical test can be performed to produce a reasonable estimation of any effect, give the low number of patients typical for this design.

## Results

### Participants

Six AD patients completed the baseline phase. One patient dropped out during the intervention phase for motivational reasons. Three males and two females successfully completed all three phases (mean age 39.3 years (*SD 11.5*) (Table 1). One patient experienced technical problems with the smart watch application. As a result, no data is available on this persons nocturnal scratching behavior.

**Table 1:** Patient characteristics.

**Participant**	**Gender**	**Age (years)**	**EASI score at T0 (interpretation)**
1	Male	41	34.8 (Severe)
2	Female	25	34.2 (Severe)
3	Male	39	22.2 (Severe)
4	Male	37	Drop out
5	Male	33	19.3 (Moderate)
6	Female	56	6.5 (Mild)

### Target measures

#### Visual analysis of day time scratching

**Figure 2:**
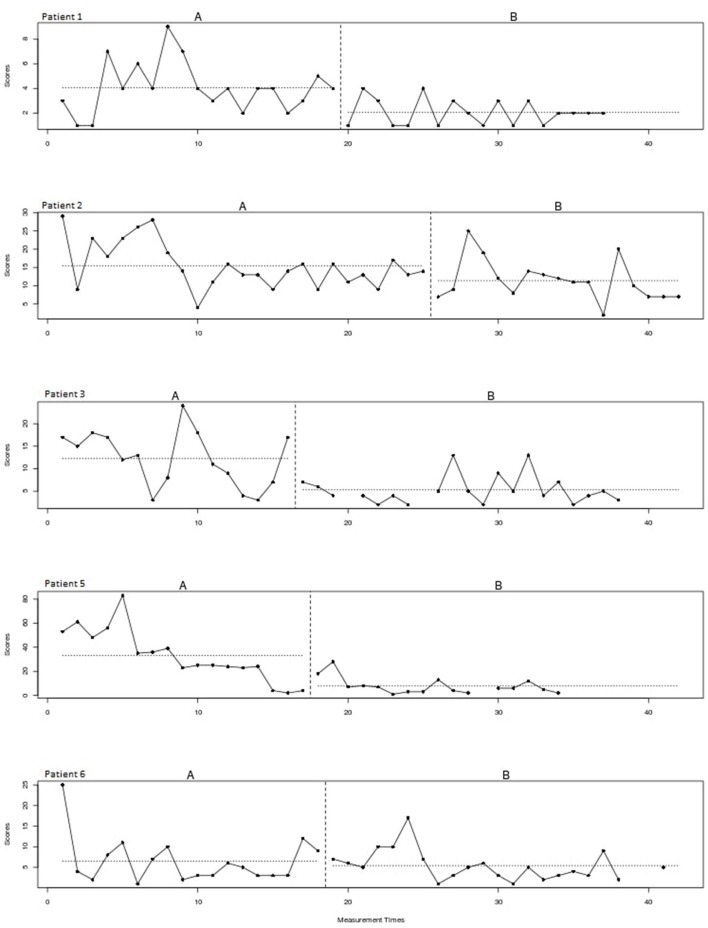
Scratching behavior of individual patients over time for the baseline **(A)** and intervention **(B)** phase.

Figure 2 shows the plots of the individual patients. The vertical dotted lines represent the end of the baseline (A) and the start of the intervention phase (B). The horizontal lines represent the mean of scratching behavior for both phases. The visual inspection of the individual patterns in Figure 2 shows a decrease in the scratching behavior for all patients after starting with the intervention. In most patients the scratching behavior fluctuated heavily and a negative trend is already visible during the baseline phase. Please note that patient 5 underwent a change of medication during the intervention phase.

#### Effect size

A non-overlap of all pairs (NAP) effect size of 0.74 is found, which indicates a moderate effect of the treatment for this type of study design.

#### Nocturnal scratching

**Figure 3:**
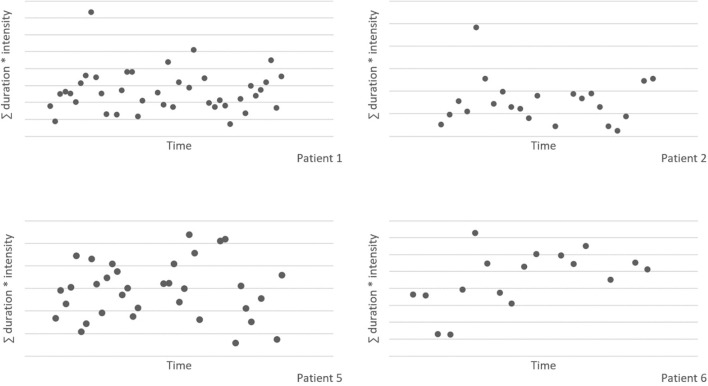
Nocturnal scratching behavior, measured with the smart watch application.

Figure 3 shows the sum of the duration and intensity of nocturnal scratching behavior for all patients, measured with the smart watch application. Due to technical problems with the smart watch application, the nocturnal scratching data of patient 3 is excluded from the figure. There appears to be no effect of the intervention on the nocturnal scratching behavior. There is a large spread, and no clear trend is visible over time.

### Standardized measures

Table 2 shows the explorative analysis of the disease activity, quality of life and self-control measures of the five patients. The results indicate that the disease activity as determined by the dermatologist (EASI) decreased between T0 and T2. Quality of life measured with the POEM questionnaire shows a large decrease between T0 and T1, with a slight increase at T2. Dermatology-specific health-related quality of life measured with the SKINDEX-17 shows a U-curve for both subscales. Furthermore, the EQ-5D-5L index and VAS scores show an increase of quality of life over time. Both factors of self-control in scratching (“positive reward” and “difficulty resisting”) decreased from T0 to T2.

**Table 2:** Explorative analysis decease activity, quality of life and self-control.

	**Median (IQR)**		
**Questionnaire** ^*^	**T0**	**T1**	**T2**
EASI	26.75 (24.95)		11.30 (8.35)
POEM	21.00 (9.25)	15.50 (9.25)	17.50 (11.50)
ZCCL “Positive reward”	8.00 (15.5)	7.50 (9.00)	6.00 (2.25)
ZCCL “Difficulty resisting”	16.00 (8.50)	12.50 (7.00)	10.00 (8.75)
Skindex-17 psychosocial	9.00 (5.50)	1.50 (1.75)	7.00 (7.00)
Skindex-17 symptoms	5.00 (1.50)	3.50 (3.25)	4.50 (1.00)
EQ-5D-5L index	0.82 (0.21)	0.83 (0.19)	0.91 (0.21)
EQ-5D-5L VAS	69.00 (17.00)	82.50 (9.00)	77.50 (20.00)

* Cut-off scores: EASI: 0 (clear); 0.1–1.0 (almost clear); 1.1-7.0 (mild); 7.1–21.0 (moderate); 21.1–50.0 (severe); 50.1–72.0 (very severe) POEM: 0–2 (clear/almost clear); 3-7 (mild); 8–16 (moderate); 17–24 (severe); 25–28 (very severe) ZCCL “Positive reward”: range from 6 to 30 ZCCL “Difficulty resisting”: range from 5 to 25 Skindex-17 Psychosocial: 0–4 (little impairment); 5–9 (moderate impairment); 10–24 (high impairment) Skindex-17 Symptoms: 0–4 (few); 5-10 (a lot) EQ-5D-5L index: range from 0 to 1 EQ-5D-5L VAS: range from 0 to 100.

### Evaluation by the patients

During the evaluation of the course of the study and the intervention, all patients indicated that they planned to continue with the learned technique in the future. Registration of the scratching did elevate the awareness of this habitual behavior and was perceived to be helpful in reducing scratching behavior. A frequently mentioned limitation of the newly learned technique is that it is difficult to apply it during daily activities. While driving a car or attending a meeting, taxing the working memory is impossible or even dangerous. Moreover, the technique was perceived to be time consuming by some of the patients. Furthermore, patients tended to “re-design” the treatment following their own preferences: some focused more on the imaginary part, and others seemed to profit more from the taxing of working memory-part of the treatment.

## Discussion

This pilot is the first study to investigate the use of the EMDR treatment protocol for urge for scratching behavior in patients with AD. Visual analysis of the data showed a decrease of scratching behavior over time in all patients. During the baseline phase, scratching behavior fluctuated heavily and already showed a slight negative trend. After receiving treatment and during follow-up, all patients showed less scratching behavior compared to the baseline registration. The NAP effect size indicated a moderate effect of the intervention. Outcomes of disease activity decreased over time and patients' self-control and quality of life improved after treatment. Furthermore, nocturnal scratching behavior did not differ after the intervention, compared to the baseline phase.

So far, little is known about the working mechanisms of the EMDR treatment protocol for urge. Initially, the effectiveness of this method in reducing scratching behavior was found by chance (12). After additional single case successes, the curiosity about the effect further rose. To learn more about possible working mechanisms, it may be useful to draw parallels with other types of unwanted behavior. For example, the use of EMDR in addictive behavior has been subject of studies in the past two decades, in smaller and larger studies, with varying results (24–26). Scratching behavior and addiction share the same sensory mechanisms and neurobiological foundations (27, 28), which makes addiction an interesting starting point in the search for explanations. Scratching is often experienced as pleasurable and can have a rewarding effect (29, 30). However, when the itch is chronic, for example in the case of AD, an itch-scratch cycle can develop: scratching provides relief in the short term, but the damage done by scratching can aggravate the itching in the long term. Also, the wounds created by scratching cause itch. This vicious circle, driven by the urge to scratch, resembles with drug addiction and share the same basic principles (31). First, scratching/intoxication serves as a positive reinforcement (high/itch relief). Second, itch returns when the scratching stops. This corresponds to withdrawal symptoms when the drug is not administered. In the third and final stage, the person gets preoccupied with the itch/drug, which results in more scratching/drug use (32). In addition to similar fundamentals, the treatments of these disorders may also show similarities (33). Most addiction-focused EMDR approaches focus on mitigating craving. Since craving in addiction shows much resemblance with the urge to scratch, the crux for successful treatment may lie in that the treatment explicitly addresses the physical component of the (addictive) behavior, for instance, reaching for a beer/cigarette or moving the scratching hand toward the skin. In this respect, the imaginary scratching shows similarities with the EMDR-technique of “cognitive interweaves,” as patients are allowed to perform the scratching in imagination, while at the same time working memory is being taxed.

A notable observation of the results is that the downward trend of the registered scratching behavior already started during the baseline phase. In other words, even before patients started with the intervention, scratching behavior already decreased. This can possibly be explained by the effect of the registration and the associated openness to change. Previous research has shown that simply registering unwanted behavior can result in a decrease in the frequency of this behavior (34, 35).

Another remarkable finding is that the smartwatch measurements show no change in nocturnal scratching behavior, while the measurements during the day showed a decrease. A conceivable explanation for this is that the intervention targets the conscious scratching behavior, but that the unconscious—and therefore automatic—scratching during the night remains the same.

Furthermore, without knowledge of the study results, most patients indicated during the evaluation that they would continue to use the learned technique in the future. Despite the mentioned limitations in using imaginary scratching, patients are willing to invest time to focus on the intervention.

### Strengths and limitations

This study is the first to investigate the effect of EMDR treatment protocol for urge on scratching behavior of AD patients in a controlled trial. To investigate this effect, high-tech methods are used to measure scratching behavior. This strength to use innovative techniques makes it possible to study all scratching episodes during day and night. However, using new high-tech measurements also entails risks. In practice, the use of the watch measurement did not always work properly.

An important limitation lies in the methodology, in particular the design of the multiple baseline. First, the Shiny application can compare only two phases. We therefore had to combine the intervention and follow-up phase. As a result, possible short-term and long-term effects of the intervention cannot be compared. Second, during the baseline, registered scratching behavior fluctuated heavily in all patients. By extending the baseline, patients are given more time to get used to the registration, which may result in a more stable course. This may result in a more reliable statements about whether the decrease of scratching behavior is attributable to the treatment. Furthermore, one patient underwent a change of medication during the intervention phase, which may have affected the results.

## Conclusion

Possible effects of treatment according to the EMDR treatment protocol for urge most likely present themselves in daily scratching, and not nocturnal scratching. The results of the visual analysis of day time scratching behavior, disease activity, quality of life, and self-control seem promising. These findings pave the way for future research into the effect of the new intervention on atopic dermatitis and other skin conditions suffering from scratching behavior, such as prurigo nodularis.

## Data availability statement

The raw data supporting the conclusions of this article will be made available by the authors, without undue reservation.

## Ethics statement

The studies involving human participants were reviewed and approved by Medical Scientific Research Ethical Committee of the Erasmus University Medical Center (reference number MEC-2020-0127). The patients/participants provided their written informed consent to participate in this study.

## Author contributions

RW-S, DD, and LK conceived and designed the study. RW-S and DH selected possible patients. MV and LK acquired, analyzed, interpreted the data, and wrote the draft. JB supervised the project. All authors critically read, reviewed and revised the manuscript, and approved the final manuscript before submission.
