# Supplementary material for: Reducing scratching behavior in atopic dermatitis patients using the EMDR treatment protocol for urge: A pilot study
Source: Front Med (Lausanne). 2023 Apr 4;10:1101935. doi: 10.3389/fmed.2023.1101935 (PMC10110898; doi:10.3389/fmed.2023.1101935)
Supplement: Supplementary file 1 [file Data_Sheet_1.DOCX]

# Appendix S1. EMDR treatment protocol for urge (Drang EMDR Protocol, DEP)

After the explanation of the treatment, rationales and an introduction of the EMDR protocol for Urge, the protocol follows six steps:

## Step 1: Place in the body

*“Close your eyes and tell me where on your body your urge to scratch is the strongest”*

(Patient names place on the body)

## Step 2: Level of Urge (LoU)

*“How strong is the urge to scratch on a scale from 0 to 10, where 0 is no urge at all and 10 is as much urge as possible”*

(Patient names a number above 0)

## Step 3: Performing the urge in imagination

*“OK, go and scratch this spot as you would like in imagination and visualize your body doing what it wants to do and follow my fingers (or the lights)”*

After about 30 seconds the taxing of the working memory stops.

## Repeat step 2: Level of Urge (LoU)

*“How strong is the urge to scratch on a scale from 0 to 10, where 0 is no urge at all and 10 is as much urge as possible”*

(With an urge higher than 0, step 3 is repeated)

## Repeat step 3: Performing the urge in imagination

*“OK, again go scratching as you would like in imagination. Visualize your body doing what you want to do and follow my fingers (or the lights)”*

After about 30 seconds the taxing of the working memory stops.

Then ask again and again *"How strong is the urge to scratch now, on a scale from 0 to 10, where 0 is no urge at all and 10 is as much urge as possible”*

Repeat this until LoU = 0

## Step 4: Whitening the spot

*“Go to the place on the body where the urge to scratch is now 0 and make it white in imagination. Then we know that you have scratched there in imagination and that place is calm and quiet now.”*

Repeat steps 1 to 4 until there is no place where the patient still feels the urge to scratch.

## Step 5: Positive closure

*“What is the most positive or valuable that you have experienced or learned about yourself or the urge during this session?”*

## Step 6: Homework and registration

*“We have reduced the urge to scratch to zero. Now I ask you to apply this method at home. Whenever you feel the urge to scratch, sit down and give in to the urge in imagination. That is, you start scratching as hard as you can or as you would like in imagination and visualize your body doing what it wants to do, while moving your fingers back and forth in front of your eyes and your eyes following your fingers"* (or another technique of taxing the working memory).

*“Once the urge has decreased to zero, you can stop until the urge comes back and apply the method again.”*

*“Experience shows that it often takes a short while before the level of urge is zero, but that the urge does come back so that you will have to repeat this method a few times until it really goes away.”*

*“Again: you have to do everything you can not to scratch in real life but only to do it in imagination. This is how you overcome the urge to scratch.”*

*“Every day you can register how often and for how long you have used the method and how many times you gave in and scratched in real life.”*

*“The next time you come, bring this registration form, so we can see whether actual scratching decreases or even no longer occurs. At some point you will no longer have to apply the method so often; but if necessary, you can always pick up the method again if the urge to scratch increases.”*

< End of protocol >

The full text version of the protocol is available on request from the corresponding author.
